# Blade of Grass as an Unusual Cause of Recurrent Facial Cutaneous Sinus Tract: A Clinical Case

**DOI:** 10.1155/2016/4639693

**Published:** 2016-06-01

**Authors:** Resmije Ademi-Abdyli, Feriall Perjuci, Teuta Bicaj, Yll Abdyli

**Affiliations:** ^1^Department of Oral Surgery, Medical Faculty, University of Prishtina, Dental Branch, 10000 Prishtina, Kosovo; ^2^Department of Prosthetic Dentistry, Medical Faculty, University of Prishtina, Dental Branch, 10000 Prishtina, Kosovo; ^3^Medical Faculty, University of Prishtina, Dental Branch, 10000 Prishtina, Kosovo

## Abstract

The presence of an embedded foreign body in the oral and maxillofacial region is not unusual, but the impaction of a foreign body with vegetative nature is rare. Prompt diagnosis and surgical removal of these foreign bodies will minimize their associated complications. This case report presents a patient with recurrent submandibular abscess and persistent facial cutaneous sinus tract caused by a retained blade of grass inside the facial soft tissue. The fact that the plain radiograph misdiagnosed the presence of a foreign body meant that the pathology persisted for about three months, and the patient underwent hospitalization, surgical procedures, and antibiotic regimens; however all of these failed until the foreign body was detected and removed.* Conclusion*. To avoid misdiagnosis of foreign body presence in the orofacial region, notably suspected foreign bodies with low radiopacity, the clinician must perform careful clinical examination and use the ultrasonography. Also, in the uncertain cases where the pathology persists, despite having undertaken surgical procedures and antibiotic regimens, the clinician should pay more attention to the patient's history which may suggest the presence of the foreign body.

## 1. Introduction

The cutaneous sinus tract in the orofacial region as a sequel of dental origin is a common condition, and its diagnosis is usually easy, if the clinician considers the possibility of its dental origin. However, cutaneous sinus tract can also be a sequel of a retained occult foreign body (FB) in soft tissues [1].

The entry of foreign bodies (FBs) into the orofacial region and their inculpation is not unusual. Their entrance could be accidental due to trauma or iatrogenic factors [2–4].

FBs may remain dormant in the soft tissue for years without causing damage to adjacent structures [1]; however, some FBs can be a source of chronic inflammatory reactions and infection [5, 6].

Although most of the impacted FBs in oral and perioral tissue are of inorganic nature such as parts of surgical instruments or burs and filling materials (amalgams, composites, cements, or gutta-percha points) [4, 7, 8], the researched literature reveals few cases with FB impaction of a vegetative nature [1, 6, 9–12].

Detection of FBs inside the soft tissue is often very difficult because the ingrained FBs induce a reparative granuloma formation which surrounds them, making their detection by eye difficult, even during surgical treatment [1]. The early detection and diagnosis of FBs is usually based on the patients' history, clinical examination, and the various radiographic imaging methods such as plain radiographs, CT (computed tomography), and ultrasound.

The visibility of FBs on plain radiographs depends on their ability to absorb X-rays, their density and the difference in density between them and the tissue in which they are embedded, therefore conventional plain radiography is helpful only in cases of radiopaque FBs.

When common radiographs, patient history and clinical examinations fail to reveal the presence of retained FBs with low radiopacity, they can be detected using CT scans and ultrasonography [1, 4, 12–18].

Based on the study of Javadrashid et al. [17], wood could only be detected using an ultrasound as it is highly sensitive and more effective than CT for identifying the superficial FBs with low radiopacity.

When the history is dubious and radiographic analyses are inconclusive, surgical exploration remains the diagnostic choice in conjunction with therapeutic modality.

## 2. The Objective of the Study

To alert clinicians that in the process of FB diagnosis, in addition to clinical, radiographic, and other available examination methods, the patient's history should be considered essential, especially in the uncertain cases where the pathology persists, despite performed surgical procedures and antibiotic regimens.

## 3. Clinical Case

A 28-year-old male reported to the Department of Oral Surgery with a history of complaints such as pain, recurrent swelling of the cheek, and cutaneous sinus tract, over a period of three months.

Based on patient's history, more than two months earlier, he attended to the Maxillofacial Surgery Department with complaints as pain and recurrent swelling of the cheek. The maxillofacial surgeon performed an extraoral incision and drainage of the pus from the submandibular region and suggested surgical extraction of the impacted wisdom tooth, documented by lateral radiography, as the cause of complaints. According to patient's data, although the oral surgeon performed the surgical removal of the wisdom tooth, he still had the same complaints. Consequently, he returned to the Department of Oral Surgery. Since a postsurgical wound infection was suspected, the oral surgeon performed the curettage of the surgical wound and prescribed antibiotics.

After a month, despite the previously performed treatments (tooth extraction, repeated tooth sockets curettages, and antibiotic regimens), the same symptoms returned, so he attended again to the Oral Surgery Department, the last dental visit. The complaints presented as dull pain, recurrent swelling, and purulent discharge from the cutaneous sinus tract at the incisional site of the involved region.

## 4. Clinical Findings and Diagnosis

**Figure 1:**
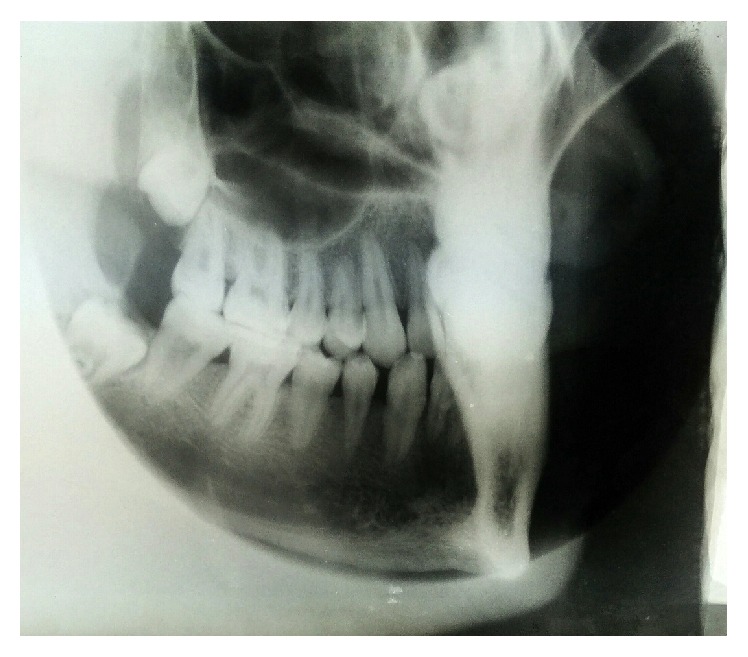
Preoperative lateral radiography of right side mandibular wisdom tooth.

**Figure 2:**
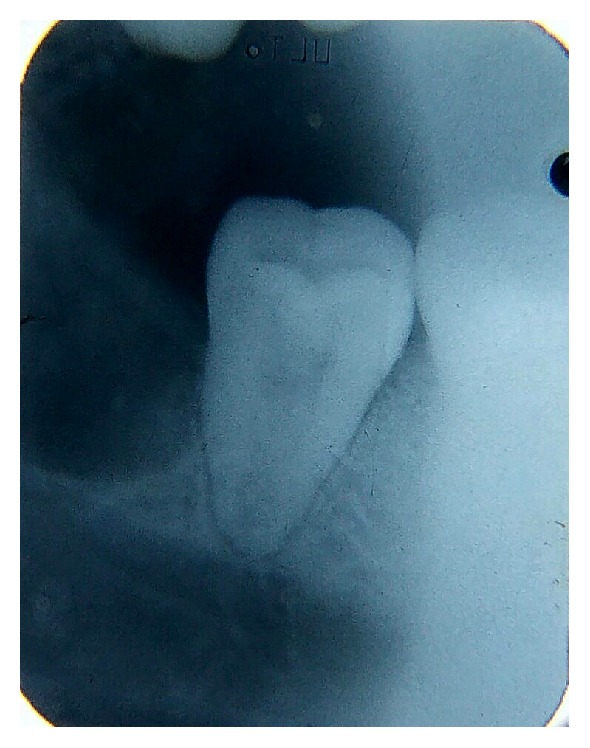
Postoperative retroalveolar radiography of postextraction tooth socket (wisdom tooth).

Upon extraoral inspection of the right submandibular region, cutaneous sinus tract covered with scrub was noted at the incisional site, resulting in purulent discharge under pressure. Intraoral examination revealed a postoperative wound (postextraction tooth socket) of the right wisdom tooth, which did not heal completely after two months of tooth extraction. The patient's past medical history, dental history, and clinical examination were not helpful for any diagnosis. Since as evidence for patient's preoperative dental situation we had at our disposal only patient's lateral radiography (Figure 1), which showed a right mandibular impacted wisdom tooth, a retroalveolar radiograph was requested (Figure 2) with the aim of searching for the cause of delayed wound healing and persistent cutaneous sinus tract.

**Figure 3:**
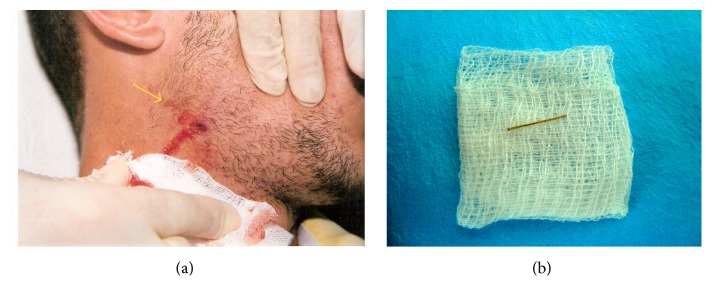
(a) Blade of grass revealed during curettage of cutaneous sinus tract. (b) Blade of grass.

As the conventional retroalveolar radiography did not reveal anything suspicious (Figure 2), it was decided to perform the revision of the postoperative tooth socket and curettage of the extraoral cutaneous sinus tract, under local anesthesia. During intraoral curettage of the tooth socket, only granulations were dislodged, while, during extraoral curettage of cutaneous sinus tract canal, a short and thin FB, similar to that of a tooth brush fiber, appeared unexpectedly (Figure 3).

 It was difficult to determine the nature of this FB, but the patient eagerly exclaimed “*It's grass. It's a blade of grass which I used to clean my tooth three months ago.*”

Upon our request for an explanation as to the way in which inoculation of this occult FB occurred, the patient recited the history of this blade of grass. The patient disclosed that, three months earlier, he had a soft tissue inflammation around the right mandibular wisdom tooth. In order to clean the pocket between the inflamed soft tissue and the tooth, he used the long blade of grass, a part of which unstrapped and disappeared somewhere in his mouth.

That same night, the first complaints of severe pain at the right side of the lower jaw began, followed by complaints of swelling of the cheek on the same side on the very next day. He visited the regional general dentist twice within two weeks. Although he used the prescribed antibiotics by general dentist, his condition was worsening, so the patient asked for help from a general practitioner who suggested hospitalization in the Department of Infectious Diseases, where he was treated for infectious parotitis for approximately ten days.

According to patient's history, he was well for almost two weeks following hospitalization, but later on the pain and swelling recurred, so he was referred to the Maxillofacial Surgery Department where he was initially diagnosed, extraoral incision was performed, and he was referred to the Oral Surgery Department for impacted tooth extraction. Despite surgical removal of the impacted wisdom tooth, he still had the same complaints; therefore, he returned to the Department of Oral Surgery several times including this last visit.

In response to why he did not mention the blade of grass when he first attended the Maxillofacial and Oral Surgery Department, the patient revealed that during his initial complaints (pain and swelling) at his first visit to the general dentist, and during hospitalization at the Clinic for Infectious Diseases, he emphasized the incident with the blade of grass but the clinicians neglected this history, making him cast away his bad experience with the blade of grass as he deemed it to be insignificant information. Following the patient's explanation about the history of the blade of grass and careful examination of the retained FB, the case was finally diagnosed as

*Recurrent facial cutaneous sinus tract of submandibular region secondary to the retained blade of grass inside the orofacial soft tissues.*

## 5. Discussion

Based on the literature, the impaction of foreign bodies (FBs) with vegetative nature is rare [1, 5, 9–11]. They can induce an intense inflammatory reaction in the surrounding tissues and may get a secondary infection, so the patient may present with recurrent, nonhealing sinus on the orofacial region [1, 4]. FBs sometimes migrate within the tissues and become symptomatic after a certain period of time. In these cases, it is very difficult to discern the direct relationship between the suspected FBs with the clinical symptoms presented [10].

Their detection is often very difficult, especially the radiolucent vegetative FBs lodged deep in the tissue, because plain radiographs are unable to detect them. These vegetative FBs can go undetected, causing significant morbidity, repeated visits, high cost, and extensive surgery if another imaging is not considered [12, 13, 19].

To avoid misdiagnosis, especially in uncertain cases where the pathology persists even after conventional management, the clinician must consider an impacted FB as a possible cause of recurrent abscess or sinus tract [9]. Therefore, the clinician should perform careful examination and pay close attention to the patient's history which may suggest the presence of the FB. Furthermore, prompt diagnosis and surgical removal of FBs will minimize their associated complications [4].

The course of our presented case show the similarity with the cases presented in the literature [4, 10]. But actual presented case related with a short, thin blade of grass, initially inoculated intraorally, which migrated extraorally within the soft tissue of the face; relatively far from its primary inoculation, complicated with pain, recurrent swelling and cutaneous sinus discharge were bigger diagnostic challenge, because of the fact that patient's history at Maxillofacial and Oral Surgery Department did not offer any data regarding FB presence. Also the inoculated vegetative FB with low density structure was impossible to visualize with disposal conventional radiographs, such as lateral and retroalveolar radiography (Figures 1 and 2)

The presented case, respectively, patient's bad experience with the misdiagnosed, retained FB inside the soft tissue of the face, its consequences, and many follow-up treatments including surgical tooth extraction, incision, drainage, antibiotic therapy, and repeated curettages, can be explained, blamed, or even somehow justified with the following facts:

1. The general dentist, general practitioner, and specialist of infectious diseases misdiagnosed the case because of erroneous taking of the patient's history and clinical examination and negligence with regard to the patient's history in relation to the problems with his wisdom tooth and his experience with the blade of grass. Thus, unfortunately, the patient was mistakenly treated for infective parotitis.
2. The maxillofacial surgeon's misdiagnosis arouses from the fact that, throughout the dental history, the patient did not offer any indication of foreign body presence, and hence the maxillofacial surgeon appointed all of the patient's complaints to the impacted wisdom tooth as the possible cause of symptoms (recurrent infection with swelling and pain). So he accordingly performed the extraoral incision and preferred surgical tooth extraction.
3. The case was also misdiagnosed at his first two visits to the Oral Surgery Department, because again the patient did not offer any indication for FB presence, and the conventional lateral radiography did not show a vegetative FB being radiolucent, so the oral surgeon performed the extraction of the impacted tooth, suspected as the cause of the patient's problems.

So on the patient's last (third) visit to the Oral Surgery Department, we were both fortunate and surprised to retrieve the blade of grass as an occult FB during the curettage of the facial sinus tract, as we did not suspect to find anything suchlike, because of wanting patients history about the inoculated blade of grass and lacking of the retroalveolar radiography to visualize the FB with low density structure. Thus finally, the case was diagnosed as “*recurrent facial cutaneous sinus tract of submandibular region secondary to the retained blade of grass inside the orofacial soft tissues*” after the removal to which the patient responded well and the cutaneous sinus tract healed uneventfully.

## 6. Conclusion

To avoid misdiagnosis of all pathologies and notably the misdiagnosis of FB presence, as well as performing clinical examinations, the clinician must pay attention to the patient's history which may be suggestive of FB presence; especially in uncertain cases where the pathology persists even after surgical procedures and antibiotic regimens. In cases where strong suspicion for a retained FB exists based on the patient's history, although not seen on a plain radiograph, the alternative route of investigation should be the use of an ultrasound or surgical exploration.

## Abbreviations

CT: Computed tomography
FB: Foreign body.

## References

[1] Auluck A., Behanan A. G., Pai K. M., Shetty C. (2005). Recurrent sinus of the cheek due to a retained foreign body: report of an unusual case. *British Dental Journal*.

[2] McKinney R. V., Brady G. L., Singh B. B. (1981). Metallic foreign body embedded in the cheek for 20 years. *The Journal of the American Dental Association*.

[3] Quiamud D. (2001). Foreign bodies in maxillofacial region. *Journal of the Pakistan Dental Association*.

[4] Aregbesola S. B., Ugboko V. I. (2013). Unusual foreign bodies in the orofacial soft tissue spaces: a report of three cases. *Nigerian Journal of Clinical Practice*.

[5] Martins W. D., Fávaro D. M., Westphalen F. H. (2005). Emergency maxillofacial radiology. Foreign body localization: report of cases. *Dentomaxillofacial Radiology*.

[6] Spengos M. N. (1977). Roentgeno-oddities: a pea in the cheek. *Oral Surgery, Oral Medicine, Oral Pathology*.

[7] Cataldo E., Santis H. (1974). Response of the oral tissue to exogenous foreign materials. *Journal of Periodontology*.

[8] Weathers D. R., Fine R. M. (1974). Amalgam tattoo of oral mucosa. *Archives of Dermatology*.

[9] Sethi A., Sabherwal A., Basu S. K., Sareen D. (2006). An unusual cause of recurrent cheek abscess: a clinical case. *Brazilian Journal of Oral Sciences*.

[10] Mohanavalli S., David J., Gnanam A. (2011). Rare foreign bodies in oro-facial regions. *Indian Journal of Dental Research*.

[11] Sobol S. E., Jacobs I. N., Levin L., Wetmore R. F. (2004). Pistachio nutshell foreign body of the oral cavity in two children. *International Journal of Pediatric Otorhinolaryngology*.

[12] Graham D. D. (2002). Ultrasound in the emergency department: detection of wooden foreign bodies in the soft tissues. *Journal of Emergency Medicine*.

[13] Oikarinen K. S., Nieminen T. M., Mäkäräinen H., Pyhtinen J. (1993). Visibility of foreign bodies in soft tissue in plain radiographs, computed tomography, magnetic resonance imaging, and ultrasound: an in vitro study. *International Journal of Oral & Maxillofacial Surgery*.

[14] Tahmasebi M., Zareizadeh H., Motamedfar A. (2014). Accuracy of ultrasonography in detecting radiolucent soft-tissue foreign bodies. *Indian Journal of Radiology and Imaging*.

[15] Manthey D. E., Storrow A. B., Milbourn J. M., Wagner B. J. (1996). Ultrasound versus radiography in the detection of soft-tissue foreign bodies. *Annals of Emergency Medicine*.

[16] Orlinsky M., Knittel P., Feit T., Chan L., Mandavia D. (2000). The comparative accuracy of radiolucent foreign body detection using ultrasonography. *The American Journal of Emergency Medicine*.

[17] Javadrashid R., Fouladi D. F., Golamian M. (2015). Visibility of different foreign bodies in the maxillofacial region using plain radiography, CT, MRI and ultrasonography: an in vitro study. *Dentomaxillofacial Radiology*.

[18] Aras M. H., Miloglu O., Barutcugil C., Kantarci M., Ozcan E., Harorli A. (2010). Comparison of the sensitivity for detecting foreign bodies among conventional plain radiography, computed tomography and ultrasonography. *Dentomaxillofacial Radiology*.

[19] Anderson M. A., Newmeyer W. L., Kilgore E. S. (1982). Diagnosis and treatment of retained foreign bodies in the hand. *The American Journal of Surgery*.

